# Numerical model for solid-like and fluid-like behavior of granular flows

**DOI:** 10.1007/s11440-024-02364-2

**Published:** 2024-07-16

**Authors:** Yadong Wang, Wei Wu

**Affiliations:** Institute of Geotechnical Engineering, Boku University, Feistmantelstraße 4, 1180 Vienna, Austria

**Keywords:** Fluid-like flow, Granular material, Hypoplastic model, Rheology, Solid-like flow

## Abstract

We propose a constitutive model for both the solid-like and fluid-like behavior of granular materials by decomposing the stress tensor into quasi-static and collisional components. A hypoplastic model is adopted for the solid-like behavior in the quasi-static regime, while the viscous and dilatant behavior in the fluid-like regime is represented by a modified $\mu (I)$ rheology model. This model effectively captures the transition between solid-like and fluid-like flows. Performance and validation of the proposed model are demonstrated through numerical simulations of element tests.

## Introduction

Granular materials may exhibit solid-like and fluid-like (including gaseous) behaviors in different flow regimes [3, 14, 38, 68]. In the quasi-static regime, the granular material behaves like an inviscid solid, where the frictional force between the particles dominates the deformation. Upon reaching the critical state and beyond, the collisional forces begin to develop. Along with elevating shear rate, in the collisional regime, the granular flow becomes more dilute and viscous, and the flow is mainly dictated by the grain collisions [14]. It is desirable to use a unified constitutive model for both flow regimes. However, bridging the solid-like and fluid-like regimes poses a major challenge for constitutive models.

For granular flow in the quasi-static regime, some plasticity models with the critical state have been developed [11, 15, 31, 34, 35, 58, 60, 67, 69]. Usually, the equivalent frictional coefficient is regarded as constant and rate-independent until the critical state is reached. Suppose that the flow maintains in the critical state, the granular flow evolves from the quasi-static regime into the intermediate flow regime, where the flow becomes more and more rate-dependent. At very high shear rate, the frictional force becomes negligible, and the transition between solid-like and fluid-like flows can be observed.

For the fluid-like regime, some experiments and discrete particle simulations indicate that the frictional coefficient and volume fraction can be related to a dimensionless inertial number [18, 25, 26, 38]. The well-known $\mu (I)$ rheology model was proposed to describe the granular flows in the intermediate regime [26]. This model has been verified for various configurations [12, 21, 24, 27, 29, 52, 53, 65]. This model was also extended to consider the “shearing dilation” behavior caused by particle collisions [5, 55]. However, one of the limitations of this local rheology model is that it is difficult to combine this model with the constitutive models in the quasi-static regime [18, 25].

Several attempts have been made to capture the transition between solid-like and fluid-like flows by combining the plastic models with rheology models [2, 6, 9, 12, 16, 19, 20, 27, 47, 51, 55]. Most models assume an apparent viscosity during the whole process. Such models can be categorized into three groups: In the first group, the local rheology model is coupled with a yielding criterion to define a visco-plastic type model [4, 12]; in the second group, granular elasticity without the Coulomb condition and rate-sensitive fluid-like flow are reformulated and combined into one universal elastoplastic law [16, 27]; in the third group, the stress is assumed to possess two separate branches distinguished by the critical volume fraction [13, 42–44, 59]. Some attempts have been made to bridge the solid-like and fluid-like flows based on the last approach [5, 10, 39, 51, 55, 57, 63]. However, most models assume strongly simplified material behavior in the quasi-static regime, which compromises their performance for the solid-like flow regime.

In this paper, we propose an improved hypoplastic constitutive model to bridge solid-like and fluid-like flows in dry granular materials. Initially, we define a yielding criterion that integrates the critical state with rate-dependent shearing for granular flows. Subsequently, the total stress throughout the shearing process is decomposed into solid-like and fluid-like components, ensuring a smooth transition between these states. Building on this foundation, we introduce a rate-dependent hypoplastic model that incorporates the critical state. Within this framework, the quasi-static and collisional stress components are described using a hypoplastic equation and a modified $\mu (I)$ model, respectively. The performance of the proposed model will be thoroughly discussed, with a focus on deconstructing rate effects and constructing the transition regime. Finally, we will present a validation example to demonstrate the efficacy of the proposed approaches.

The sections of this paper are organized as follows: Section 2 shows the mathematical derivations of unified yielding criterion and constitutive model. This section also reviews the formulation of the hypoplastic model, $\mu (I)$ model, and the approaches to bridge the solid-like and fluid-like flows. Section 3 details the performance of the proposed model. Section 4 provides a validation example for the proposed model.

## Constitutive model

### General aspects

Dry granular materials exhibit distinct behaviors resembling solids and fluids across various flow regimes. Building upon previous studies [13, 14, 59], the total effective stress can be decomposed into quasi-static and collisional components, yielding:

1 $$\begin{aligned}&\varvec{\upsigma } = \varvec{\upsigma }^q + \varvec{\upsigma }^c \end{aligned}$$

in which the subscripts *q* and *c* represent the quasi-static (solid-like) and collisional (fluid-like) components of a field variable in the whole paper; $\varvec{\upsigma } = \textbf{s} - p \textbf{I}$ stands for the Cauchy stress tensor; $\textbf{s}$ represents the deviatoric stress tensor; *p* is the total pressure; $\textbf{I}$ is the identity tensor. Note that, in this paper, the definition of $\varvec{\upsigma }^c$ follows the description presented by Savage and Sayed [54]. The collisional stresses are induced by the collisional transfer of momentum and energy during nearly instantaneous collisions.

**Fig. 1:**
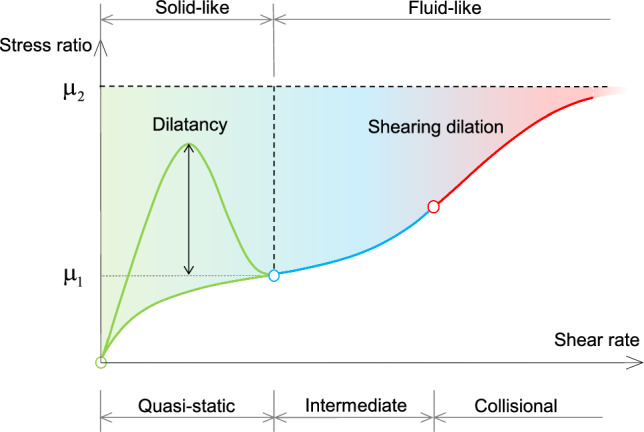
Evolution of the equivalent frictional coefficient evolves with the shear rate, where $\mu _1$ and $\mu _2$ are lower and upper limitations of $\mu$, respectively

Referring to Fig. 1, solid-like flows exhibit distinctive characteristics, such as dilatancy and static liquefaction, primarily influenced by the internal friction force among particles. These characteristics remain rate-independent and govern the stress ratio $\tau / p$, where $\tau = \sqrt{\textbf{s}:\textbf{s} /2}$, reflecting the impact of dilatancy strength and residual strength [58]. On the other hand, fluid-like flows, particularly in extreme fast flow regimes, undergo the phenomenon of “shearing dilation” [3]. As the shear rate increases, the solid fraction $\Phi$ decreases, and there is a simultaneous dependence of the pressure on the shear rate [18]. In this stage, the stress ratio is bounded by its lower and upper limitations, $\mu _1$ and $\mu _2$, respectively. An effective model should encompass these essential properties exhibited by both solid-like and fluid-like flows. To describe the features mentioned above, constitutive relationships for the solid-like and fluid-like stress components are established by assuming two constitutive equations for the quasi-static and collisional stress components:

2 $$\begin{aligned}&\dot{\varvec{\upsigma }}^q = \textbf{H}(\varvec{\upsigma }^q, \dot{\varvec{\upvarepsilon }}, \Phi ) \end{aligned}$$

3 $$\begin{aligned}&\varvec{\upsigma }^c= \textbf{D}(\varvec{\upsigma }^q, \varvec{\upsigma }^c, \dot{\varvec{\upvarepsilon }}, \mu ,\Phi ) \end{aligned}$$

where $\textbf{H}$ and $\textbf{D}$ are tensorial functions; $\dot{\varvec{\upvarepsilon }}=\left[ \nabla \textbf{v} + (\nabla \textbf{v})^\textrm{T}\right] / 2$ represents the strain rate tensor; $\Phi$ is the solid volume fraction; $\mu$ is the frictional coefficient, which depends on the strain rate. The subsequent sections of this paper aim to utilize the quasi-static stress ($\varvec{\upsigma }^q$) and collisional stress ($\varvec{\upsigma }^c$) to describe the rate-independent and rate-dependent behaviors of granular materials, respectively.

### Yielding criterion

The yielding criterion is formulated based on the equivalent shear stress $\tau$ as a function of the total effective pressure *p* via the equivalent frictional coefficient $\mu$ [55], represented by the following equation:

4 $$\begin{aligned}&\tau = \mu p \end{aligned}$$

Following the framework outlined in Sect. 2.1, the total pressure is decomposed into two branches involving quasi-static component $p^q$ and collisional component $p^c$ as [13]

5 $$\begin{aligned} p = p^q + p^c \end{aligned}$$

herein $p^q = - \textrm{tr}\varvec{\upsigma }^q / 3$, where the operator $\mathrm tr$ means the trace of a tensor. It is important to note that $p^c > 0$ is activated only when the solid volume fraction $\Phi$ is less than a critical value $\Phi _c$; otherwise, $p^c$ is enforced to be zero.

**Fig. 2:**
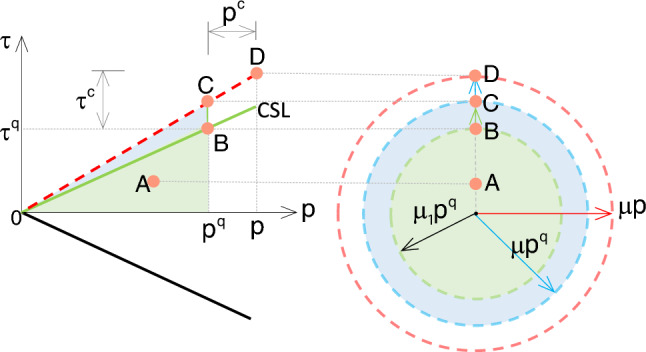
Schematic of the yielding criterion with a certain shear rate, where CSL means the critical state line

By substituting Eq. (5) into Eq. (4), Eq. (4) can be written out as

6 $$\begin{aligned} {\begin{matrix} \tau = \mu _1 p^q + \left( \mu - \mu _1 \right) p^q + \mu p^c \end{matrix}} \end{aligned}$$

herein $\mu _1$ and $\mu _2$ are lower and upper limitations of equivalent frictional coefficient $\mu$, respectively. Normally, the value of $\mu _1$ can be defined as $\mu _1 = \tan \phi$, where $\phi$ is the internal friction angle of granular material. Based on Eq. (6), the stress states in various flow regimes for a specific shear rate are shown in Fig. 2. The figure includes the critical state line (CSL), while the red dashed line represents the modified evolving yielding criterion based on the shear rate and $\Phi$. The blue line corresponds to the red line but without considering “shearing dilation.” $\tau ^q$ and $\tau ^c$ are quasi-static and collisional components of shear stress corresponding to $\varvec{\upsigma }^q$ and $\varvec{\upsigma }^c$, respectively. Various stress states are presented by the following points:

- Point A represents the initial stress state.
- Point B stands for the stress state at the critical state without the rate effect, defined by the classical yielding criterion $\tau = \mu _1 p^q$ [58, 62].
- Point C denotes the yielding criterion $\tau = \mu p^q$, which can be used to describe the fluid-like flows with $\nabla \cdot \textbf{v} = 0$, and satisfies the condition $p^c\ll p^q$ [2, 4].
- Point D lies in the intermediate regime where $\tau$, *p*, and $\Phi$ evolve with shear rate. Its yielding criterion is formulated as $\tau = \mu (p^q + p^c)$ [4].

### Model in solid-like flows

In the quasi-static flow regime, the dilatancy effect vanishes gradually as the granular materials approach the critical state, leading to the occurrence of isochoric shearing. To describe the solid-like flows of granular material up to the critical state, the hypoplastic model is employed. Building upon the classical yielding criterion (Point B in Fig. 2), the generalized hypoplastic model can be expressed as follows [62]:

7 $$\begin{aligned}&\mathring{\varvec{\upsigma }}^q = f_s \left[ \textrm{tr}\varvec{\upsigma }^q \dot{\varvec{\upvarepsilon } } + f_v \textrm{tr}\dot{\varvec{\upvarepsilon }} \varvec{\upsigma }^q + f_a^2 \frac{\textrm{tr}(\varvec{\upsigma }^q \cdot \dot{\varvec{\upvarepsilon }})}{\textrm{tr}\varvec{\upsigma }^q} \varvec{\upsigma }^q + f_e f_a (\varvec{\upsigma }^q + \textbf{s}^q) \Vert \dot{\varvec{\upvarepsilon }} \Vert \right] \end{aligned}$$

8 $$\begin{aligned}&\dot{\varvec{\upsigma }}^q = \mathring{\varvec{\upsigma }}^q + \dot{\varvec{\upomega }}\cdot \varvec{\upsigma }^q-\varvec{\upsigma }^q\cdot \dot{\varvec{\upomega }} \end{aligned}$$

9 $$\begin{aligned}&\dot{e} = (1+e)\textrm{tr}\dot{\varvec{\upvarepsilon }} \end{aligned}$$

where $\mathring{\varvec{\upsigma }}^q$ is the Jaumann stress rate; $\textbf{s}^q = \varvec{\upsigma }^q + p^q \textbf{I}$ represents the solid-like deviatoric stress; $\Vert \dot{\varvec{\upvarepsilon }} \Vert = \sqrt{ \textrm{tr}\dot{\varvec{\upvarepsilon }}^2}$ denotes the Euclidean norm; $\dot{\varvec{\upomega }} =\left[ \nabla \textbf{v} - (\nabla \textbf{v})^\textrm{T}\right] / 2$ is the spin rate tensors; $e = 1 / \Phi -1$ stands for the void ratio; $f_s$, $f_v$, and $f_a$ are functions of total stiffness, volumetric, friction angle, and relative density, respectively. The aforementioned three interpolation functions are constructed using the following parameters: initial shear modulus $E_i$, Poisson’s ratio $\nu _i$, and friction angle $\phi$, where the subscript *i* stands for “initial.” The mathematical derivations of Eq. (7) and the definitions of the related variables are provided in Appendix. In addition, $f_e$ is the function of relative density, which is given as

10 $$\begin{aligned}&f_e = \left( \frac{e-e_{min}}{e_c-e_{min}} \right) ^ {\beta _e} \end{aligned}$$

where $e_{min}$ is the minimum void ratio; $\beta _e$ is the dilatancy parameter. To describe the critical state, the void ratio at critical state $e_c$ is given as the function of $p^q$, which reads as [61]

11 $$\begin{aligned} e_c = e_{\Gamma } \exp \left[ -\zeta \left( \frac{p^q}{p_0} \right) ^\xi \right] \end{aligned}$$

in which $e_{\Gamma }$ is a material constant, which presents the value of $e_c$ for $p^q = 0$; $\zeta$ is a constant to reduce the sensitivity of $f_e$, and its value is fixed as 0.12 in this paper; $p_0 \approx 101.325$ kPa is the atmospheric pressure; $\xi$ is a parameter to control the evolution of $e_c$, and its value can be obtained by fitting together with $\beta _e$ by trial-and-error method.

Finally, the quasi-static stress components can be calculated as follows:

12 $$\begin{aligned} \varvec{\upsigma }^q = \int \left( \mathring{\varvec{\upsigma }}^q + \dot{\varvec{\upomega }}\cdot \varvec{\upsigma }^q-\varvec{\upsigma }^q\cdot \dot{\varvec{\upomega }}\right) \textrm{d}t \end{aligned}$$

The equations in this section describe the behavior of the rate-independent stress components in granular materials until reaching the critical state, taking into account the internal friction angle $\phi$, and the relationship between $\Phi$ and $\varvec{\upsigma }^q$.

### Model in fluid-like flows

Once the granular material reaches the critical state, the inclusion of rate-dependent viscous stress becomes necessary to uphold the momentum conservation of the particles [46]. The viscous shear stress, denoted as $\tau ^c$, is scaling with *p* via frictional coefficient $\mu$. This relationship can be expressed as [5, 26]

13 $$\begin{aligned}&\tau ^c = (\mu -\mu _1) p^q + \mu p^c \end{aligned}$$

14 $$\begin{aligned}&\mu = \mu _1 + \frac{\mu _2 - \mu _1}{I_0 + I}I \end{aligned}$$

15 $$\begin{aligned}&I = \frac{2 d \dot{\gamma }}{\sqrt{p / \bar{\rho }}} \end{aligned}$$

herein $I_0$ is the reference shear rate; *d* stands for the mean particle diameter, and $\bar{\rho }$ denotes the intrinsic density; $\dot{\gamma }$ stands for the shear rate, which is given as

16 $$\begin{aligned} \dot{\gamma } = \sqrt{\frac{\textbf{e}:\textbf{e}}{2}} \end{aligned}$$

where $\textbf{e} = \dot{\varvec{\upvarepsilon }} - \textrm{tr}\dot{\varvec{\upvarepsilon }} \textbf{I} /3$ is the deviatoric strain rate.

Equations (13–15) govern the evolution of collisional stress. Meanwhile, the expansion of the materials depends on solid fraction $\Phi$ as well as on the mean pressure *p*, which is defined as [18, 51]

17 $$\begin{aligned}&\Phi = \Phi _c - \Delta \Phi I \end{aligned}$$

where $\Phi _c$ denotes the random close packing density; $\Delta \Phi$ is the dynamic loosening factor. The characteristics of these rheological parameters can be found in previous study [51]. Note that $p \approx p^c$ is evident once the shear rate is large enough. By substituting Eq. (17) into Eq. (15), the expression for $p^c$ is given as [55]

18 $$\begin{aligned}&p^c= \bar{\rho } \left( 2 d \dot{\gamma } \frac{\Delta \Phi }{\Phi _c - \Phi } \right) ^2 \end{aligned}$$

The above equation is applicable when $\Phi < \Phi _c$. The possibility of steady flow or a jammed state for $\Phi \ge \Phi _c$ is discussed in the previous works [8, 55].

Based on the alignment condition, the viscous shear stress is satisfied by the following condition as

19 $$\begin{aligned} \frac{\varvec{\upsigma }^c + p^c \textbf{I}}{\left( \mu - \mu _1 \right) p^q + \mu p^c} = \frac{\textbf{e}}{ \dot{\gamma }} \end{aligned}$$

which leads to the expression

20 $$\begin{aligned} \varvec{\upsigma }^c= \frac{\left( \mu - \mu _1 \right) p^q }{ \dot{\gamma }} \textbf{e} +\frac{\mu p^c}{ \dot{\gamma }} \textbf{e} - p^c\textbf{I} \end{aligned}$$

The equations in this section describe the behavior of the rate-dependent collisional stress in granular materials under the condition that $\Phi < \Phi _c$, taking into account the frictional coefficient $\mu$, the shear rate $\dot{\gamma }$, and the relationship between $\Phi$ and $\varvec{\upsigma }^c$.

### Improved hypoplastic model

By combining the constitutive equations in Sects. 2.3 and 2.4, the total effective stress tensor with compressible rate-dependent components can be defined as

21 $$\begin{aligned} \varvec{\upsigma }&= \underbrace{ \int \left( \mathring{\varvec{\upsigma }}^q -\varvec{\upsigma }^q\cdot \dot{\varvec{\upomega }} + \dot{\varvec{\upomega }}\cdot \varvec{\upsigma }^q \right) \textrm{d}t }_{rate-independent} \nonumber \\&\quad + \underbrace{ \left[ \frac{\left( \mu - \mu _1 \right) p^q}{ \dot{\gamma }} + \frac{\mu p^c}{ \dot{\gamma }} \right] \textbf{e} - p^c\textbf{I} }_{rate-dependent} \end{aligned}$$

By rewriting Eq. (21), an improved hypoplastic constitutive model for bridging solid-like and fluid-like flows in dry granular materials can be expressed as

22 $$\begin{aligned} \varvec{\upsigma } =&\int f_s \left[ {\textrm{tr}}\varvec{\upsigma }^q \dot{\varvec{\upvarepsilon } } + f_v \textrm{tr}\dot{\varvec{\upvarepsilon }} \varvec{\upsigma }^q + f_a^2 \frac{\textrm{tr}(\varvec{\upsigma }^q \cdot \dot{\varvec{\upvarepsilon }})}{\textrm{tr}\varvec{\upsigma }^q} \varvec{\upsigma }^q + f_e f_a (\varvec{\upsigma }^q + \textbf{s}^q) \Vert \dot{\varvec{\upvarepsilon }} \Vert \right] \textrm{d}t \\&+\int \left( \dot{\varvec{\upomega }}\cdot \varvec{\upsigma }^q -\varvec{\upsigma }^q\cdot \dot{\varvec{\upomega }} \right) \textrm{d}t +\frac{\left( \mu - \mu _1 \right) p^q}{ \dot{\gamma }} \textbf{e} + \left( \frac{\mu }{ \dot{\gamma }} \textbf{e} -\textbf{I} \right) p^c \end{aligned}$$

Furthermore, to mitigate numerical instability, the term $1/\dot{\gamma }$ can be substituted with *m* or $\left[ 1- \exp \left( -m \dot{\gamma } \right) \right] /\dot{\gamma }$ [45, 48]. *m* serves as a normalized parameter, and its value is fixed as 100.0 in this paper. Additionally, the solid fraction can be updated using the equation $\dot{e} = (1+e) \textrm{tr}\dot{\varvec{\upvarepsilon }}$, which can be equivalently expressed as $\dot{\Phi } = -\Phi \textrm{tr}\dot{\varvec{\upvarepsilon }}$.

### Remarks

- **Remark 1:** The yielding criterion (6), which incorporates both the critical state and rate-dependent shearing, is proposed to establish the constitutive relationship. By starting from the static state, the stress of dry granular material evolves toward the yielding state (6). This approach allows for the construction of a unified constitutive model that bridges the gap between solid-like and fluid-like flows.
- **Remark 2:** It is evident from the proposed constitutive equation (22) that the solid-like and fluid-like stress components coexist. Depending on the values of parameters, different scenarios arise. When $p^c= 0$ and $\mu =\mu _1$, Eq. (22) retains the original hypoplastic model. If $p^c=0$ and $\mu \ne \mu _1$, Eq. (22) can be used to describe the material deformation with viscous effect. When $p^c> 0$ and $\mu > \mu _1$, Eq. (22) is used to describe the development of granular materials in various flow regimes.
- **Remark 3:** Based on our simulations, the unified framework demonstrates the capability to integrate hypoplasticity with other rheological models, such as the Herschel–Bulkley–Papanastasiou model [30, 36, 64] and the model proposed by Bagnold [3]. In addition, it can be observed that, in Eq. (22), the proposed constitutive equations for the solid and fluid regimes are formulated and applied separately. This rate-dependent hypoplastic constitutive model applies to the entire process from solid-like to fluid-like behavior. Similar treatments can be found in previous studies [13, 51, 57, 59].

## Performance of the proposed model

**Table 1:** Parameters for element tests

	Description	Parameter	Unit	Value
Basic parameter	Initial shear modulus	$E_i$	$\textrm{mPa}$	20.0
	Initial Poisson’s ratio	$\nu _i$	–	0.235
	Friction angle	$\phi$	$^{\circ }$	23.0
	Material constant	$e_{\Gamma }$	–	0.91
	Minimum void ratio	$e_{d}$	–	0.56
	Fitting coefficient	$\xi$	–	0.70
	Dilatancy parameter	$\beta _e$	–	1.0
Rheology parameter	Density	$\bar{\rho }$	$\mathrm{kg/m^3}$	1000.0
	Dynamic frictional coefficient	$\mu _2$	–	0.5774
	Reference shear rate	$I_0$	–	0.28
	Random close packing density	$\Phi _c$	–	0.62
	Dynamic loosening factor	$\Delta \Phi$	–	0.29
	Mean particle diameter	*d*	$\mathrm mm$	1.0

In this section, a series of undrained simple shear tests is conducted to evaluate the performance of the proposed model (22) across regimes ranging from solid-like to fluid-like flows. Given the multiple parameters involved in this study, a concise overview of the parameter calibration process is provided as follows:

- Basic material parameters such as $E_i$, $\nu _i$, $\phi$, $e_{\Gamma }$, $e_{min}$, $\bar{\rho }$, *d*, and the initial volume fraction $\Phi$ are determined through element tests.
- Fitting parameters associated with the critical state, specifically $\zeta$, $\xi$, and $\beta _e$, are derived using the trial-and-error method based on the stress–strain curves in the quasi-static regime [28, 32, 40, 60].
- Rheological parameters are ascertained through simple plane shear tests or discrete-element-method (DEM) simulations [5, 26, 38].

Consequently, the parameter values for the examples in this section are detailed in Table 1. It should be noted that the random close packing density $\Phi _c$ is initially determined using experimental data from loosely packed samples. The fitting parameters $\zeta$, $\xi$, and $\beta _e$ are subsequently derived using the trial-and-error method based on data from medium-dense samples. In this research, experimental data obtained from small polystyrene beads are utilized to complete the calibration [54].

### Rate effects

**Fig. 3:**
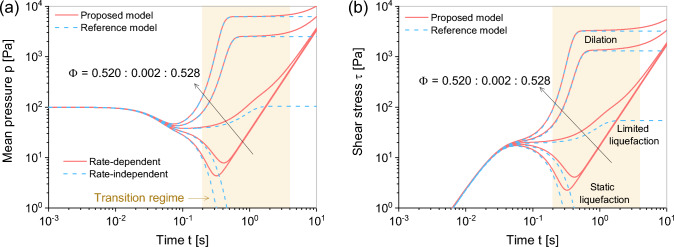
Performance of the proposed model (22) and reference model (7) in undrained simple shear test with solid fractions $\Phi = 0.520--0.528$: in accelerated flows: **a** simulation time versus mean pressure and **b** simulation time versus shear stress

**Fig. 4:**
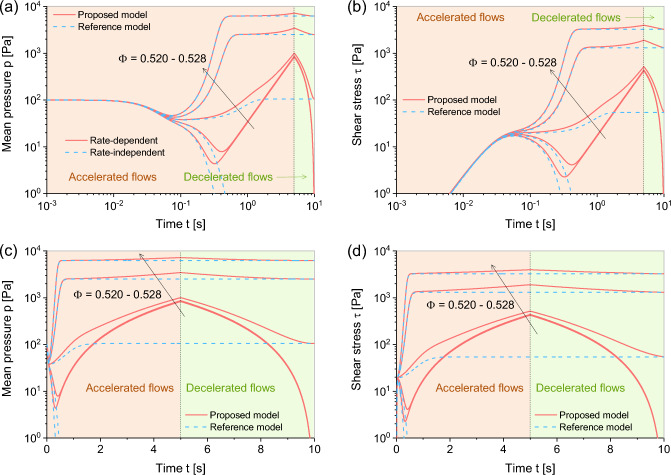
Performance of the proposed model (22) and reference model (7) in undrained simple shear test with solid fractions $\Phi = 0.520--0.528$: in accelerated and decelerated flows: (**a**, **c**) simulation time versus mean pressure and (**b**, **d**) simulation time versus shear stress

Figure 3 shows the efficacy of the proposed model (22) in undrained simple shear tests across a range of solid fractions ($\Phi = 0.520-0.528$), conducted under an initial confining pressure of 100 Pa. During these tests, strain rates $\dot{\upvarepsilon }_{xz}$, where *x* and *z* denote Cartesian coordinates, are systematically increased in a step-like fashion. Results from the proposed model are depicted with solid lines, offering a contrast to the dashed lines that represent outcomes from the reference model (7), facilitating comparative analysis. In addition, in the simulations, $t = \int \Delta t$ stands for the simulation time, with $\Delta t = 1 \times 10^{-5}$ second signifying the integration step length.

Dilation phenomena, as captured by the reference model (7), are evident in dense samples (i.e., $\Phi > 0.524$), whereas loose samples (i.e., $\Phi <0.524$) primarily exhibit static liquefaction behavior. In medium-dense sample at $\Phi = 0.524$, limited liquefaction is observed, suggesting that the materials maintain the residual stress $\varvec{\upsigma }^q$ in the fluid-like state. For all samples with various initial solid fractions, the stresses evolve to distinct constant values corresponding to the critical state, where the granular materials undergo the isochoric shearing.

Contrasting these findings, the proposed model (22) exhibits a more comprehensive response by demonstrating continuous evolution both before and after the yielding states in granular materials. For insurance, in loose samples, the proposed model successfully captures the fluid-like behavior after static liquefaction, while the reference model fails to present this phenomenon. This indicates that neglecting the collisional stress component $\varvec{\upsigma }^c$ leads to an inability to accurately describe the fluid-like behavior in this case. These observations show that the characteristics of the classical hypoplastic model have been preserved in proposed model, which additionally offers a notable enhancement in the depiction of rate-dependent residual strength.

Referring to Fig. 4, the performance of proposed model in decelerated flows is rigorously evaluated. In these simulations, the shear rate $\dot{\upvarepsilon }_{xz}$ linearly increases and subsequently decreases after $t > 5$ seconds, eventually reaching zero. The simulation results prior to 5 s align with those presented in Fig. 3. Beyond this time point, the noticeable reduction in residual strength as the shear rate declines accurately represents the deceleration of flows within the material. These observations effectively integrate critical state behavior with rheological dynamics, presenting a coherent analysis of the material response under varying conditions.

The comprehensive analysis provided through Figs. 3 and 4 demonstrates the successful integration of the rate effect within the hypoplastic constitutive relationship. This underscores the robust capability of the proposed model to accurately describe both accelerated and decelerated flows, highlighting its broad applicability in predicting complex behaviors within granular materials.

### Transition regime

**Fig. 5:**
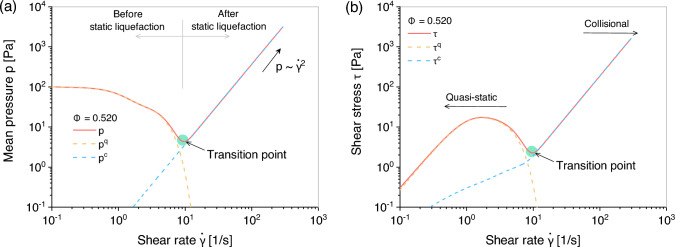
Performance of the proposed model (22) in undrained simple shear test with solid fraction $\Phi = 0.520$: **a** shear rate versus mean pressure and **b** shear rate versus shear stress

**Fig. 6:**
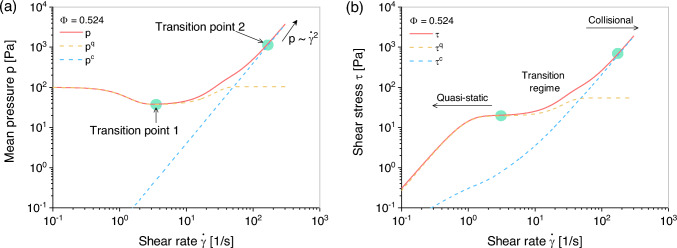
Performance of the proposed model (22) in undrained simple shear test with solid fraction $\Phi = 0.524$: **a** shear rate versus mean pressure and **b** shear rate versus shear stress

In the last section, Fig. 3 presents that dense samples, behaving like viscid fluids, require a very high shear rate for flow initiation. Typically, flow initiation is more readily achieved in loose or medium-dense samples [22]. Therefore, this section focuses on discussing samples with $\Phi = 0.520$ and $\Phi = 0.524$.

Figure 5 shows the evolution of normal and shear stress components with increasing shear strain rate in loose samples, specifically $\Phi = 0.520$. During the quasi-static regime, static liquefaction is observed, and deformation is predominantly governed by $\varvec{\upsigma }^q$. Notably, both mean pressure and shear stress display distinctive hook-like changes at the “Transition point,” marking the transition from solid-like to fluid-like flows. Upon reaching the critical state, the collisional part $\varvec{\upsigma }^c$ accumulates as the shear rate increases, resulting in rate-dependent behavior. Previous studies [1, 3, 13] have noted that once the collisional flow regime is reached, the total effective stress can be characterized by a quadratic function of the shear rate, $\dot{\gamma }$, a relationship that exemplifies the phenomenon known as “Bagnold scaling” in fully developed flow.

For medium-dense samples, such as $\Phi = 0.524$, an evident transition regime is highlighted by “Point 1” and “Point 2” as shown in Fig. 6. This is attributed to the residual frictional force between interlocked particles, resulting in stress that is proportional to the shear rate raised to a power less than two. This phenomenon indicates that frictional and collisional forces simultaneously dominate the shearing process in the intermediate regime. Consequently, it is evident that the material experience three different flow regimes including quasi-static, intermediate, and collisional regimes. In whole shearing process, the total effective stress evolves from $\lim _{\frac{p^c}{p ^q + p^c} \approx 0}\left( \varvec{\upsigma } ^q + \varvec{\upsigma }^c\right) \approx \varvec{\upsigma }^q$ to $\lim _{\frac{p^q}{p ^q + p^c} \approx 0}\left( \varvec{\upsigma } ^q + \varvec{\upsigma }^c\right) \approx \varvec{\upsigma }^c$. These observations indicate that the proposed model is a valuable component in numerical simulations for predicting the transition between solid-like and fluid-like flows in granular material.

## Model validation

**Fig. 7:**
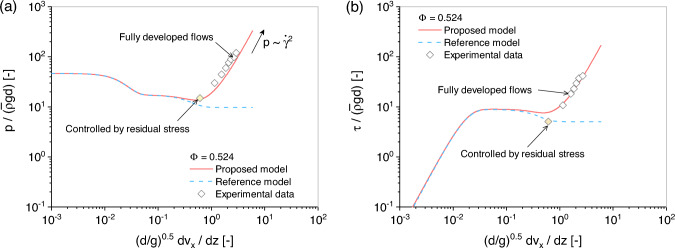
Comparison between the simulation results obtained by the proposed model (22) and reference model (7) with solid fraction $\Phi = 0.523$: **a** normalized shear rate versus mean pressure and **b** normalized shear rate versus shear stress. The markers denote experimental data from Savage and Sayed [54]

**Fig. 8:**
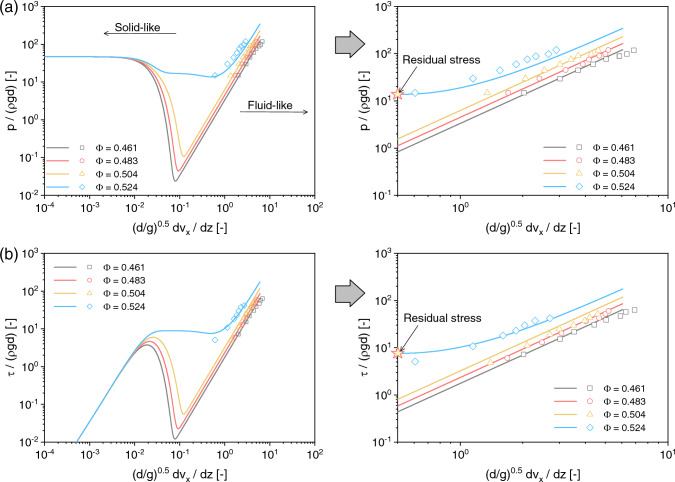
Comparison between the simulation results and experimental data: **a** shear rate versus mean pressure and **b** shear rate versus shear stress. The markers denote experimental data from Savage and Sayed [54]

To investigate the rate-dependent characteristics of granular flows, several annular shear tests on dry granular materials have been carried out by Savage and Sayed [54]. These tests can be regarded as undrained simple shearing test since the volume of the specimens remains constant. In the experiments, the initial normal stress ranges from 100 to 1500 Pa. For our model (22), a value of 500 Pa is selected as the initial confining pressure to align with the experimental conditions. The experiments with an initial volume fraction $\Phi$ ranging from 0.461 to 0.524 are used for predictions in this study. All other material parameters used in the predictions are taken from Table 1. During the simulation, the shear rate is increased in a step-like fashion, as previously mentioned. This approach corresponds to the shear rate applied in the experiments conducted by Savage and Sayed [54].

Initially, simulations were conducted on polystyrene beads with a selected solid fraction of $\Phi = 0.524$. This solid fraction was chosen because the solid particles are likely interlocked at this density, affecting the data with both frictional and collisional forces. Consequently, the original experiments with this solid fraction showcased both solid-like and fluid-like flows in granular material [54]. A comparison of simulation results obtained by the proposed model (22) and the reference model (7) is presented in Fig. 7. It can be observed that while the reference model primarily addresses material deformation influenced by frictional forces, this approach does not succeed in accurately predicting stress evolution in fully developed flows. In contrast, the proposed model achieved satisfactory predictions that align well with the experimental data.

The second set of results, presented in Fig. 8, shows a comparison between the simulation results and experimental data. Generally, the simulation results obtained by the proposed model are in good agreement with the experimental data. In the case of loose specimens, i.e., $\Phi = 0.461,0.483,0.504$, the quasi-static stress diminishes as the shear rate increases, while the development of the collisional stress is accurately reproduced. For the medium-dense case with $\Phi = 0.524$, both quasi-static and collisional stresses significantly contribute to the material deformation. As pointed out by Savage et al. [54], a higher contribution increases the likelihood of enduring contact between particles, indicating the need to consider the effects of solid fraction on residual strength. This is manifested as limited liquefaction in the intermediate flow regime, as shown in Fig. 7. Consequently, the proposed model, by combining critical state and rheological effects, successfully accounts for these features and accurately depicts the behaviors of granular materials in both solid-like and fluid-like flow regimes.

## Conclusions

In this paper, we introduce a rate-dependent yielding criterion that incorporates the critical state concept. Building upon this criterion, an improved hypoplastic model is developed to describe the evolution of the total effective stress throughout the different flow regimes. Our model assumes that the total stress can be decomposed into a quasi-static and a collisional components. The quasi-static stress is governed by a rate-independent hypoplastic constitutive equation, while the collisional stress is described by a modified $\mu (I)$ model. Through drained triaxial compression tests, we demonstrate that our model successfully captures some salient features such as dilatancy, rate effect, and deceleration flow of granular material. Moreover, the model exhibits a smooth transition between solid-like and fluid-like flows in simple shear tests. Our model has the potential to describe the deformation of granular materials both before and after liquefaction. By comparing the simulation results with experimental data, the proposed model is shown to capture the stress evolution during quasi-static, intermediate, and collisional flow regimes.

## Appendix

The flows observed in the quasi-static regime of granular materials are primarily characterized by the frictional contact between individual particles. To describe this rate-independent behavior, various elastoplastic models have been proposed to characterize the stress–strain relationship with critical state in quasi-static flow regime [31, 34, 35, 58, 67, 69]. These models utilize yield surface, plastic potential, and decomposition of deformation to capture the critical state. In contrast, we employ the hypoplastic model with critical state, which reads as [59, 60, 62]

23 $$\begin{aligned} \mathring{\varvec{\upsigma }}^q = \mathscr {L} : \dot{\varvec{\upvarepsilon }} + f_e\textbf{N} \Vert \dot{\varvec{\upvarepsilon }} \Vert \end{aligned}$$

where $\mathscr {L}$ and $\textbf{N}$ are the fourth- and second-order tensors of stress, respectively; $f_e$ equals 1 after reaching the critical state [60]. The flow rule can be derived by substituting $\mathring{\varvec{\upsigma }}^q = \textbf{0}$ into Eq. (23), which gives

24 $$\begin{aligned} \frac{ \dot{\varvec{\upvarepsilon }} }{\Vert \dot{\varvec{\upvarepsilon }} \Vert }=- \mathscr {L}^{-1} :\textbf{N} \end{aligned}$$

Taking the norm of both sides of Eq. (24), the yielding function of hypoplastic model is obtained, resulting in

25 $$\begin{aligned} \Vert \mathscr {L}^{-1} :\textbf{N} \Vert -1=0 \end{aligned}$$

Based on the framework of Eqs. (23–25), several hypoplastic models have been developed [7, 23, 37, 41, 60]. The following applications show the capability of hypoplastic model in predicting the behavior of granular materials [17, 33, 49, 50, 56, 66]. In this paper, the following hypoplastic constitutive equation is adopted [62]:

26 $$\begin{aligned} \mathring{\varvec{\upsigma }}^q = c_1\textrm{tr}\varvec{\upsigma }^q \dot{\varvec{\upvarepsilon } } + c_2 \textrm{tr}\dot{\varvec{\upvarepsilon }} \varvec{\upsigma }^q + c_3 \frac{\textrm{tr}(\varvec{\upsigma }^q \cdot \dot{\varvec{\upvarepsilon }})}{\textrm{tr}\varvec{\upsigma }^q} \varvec{\upsigma }^q + c_4 f_e (\varvec{\upsigma }^q + \textbf{s}^q) \Vert \dot{\varvec{\upvarepsilon }} \Vert \end{aligned}$$

where $c_1$, $c_2$, $c_3$, and $c_4$ are material parameters, which can be related to Young’s modulus $E_i$, Poisson’s ratio $\nu _i$, dilatancy angle $\psi$, and internal friction angle $\phi$ [19, 37, 40]. Thus, Eq. (26) can be written out as

27 $$\begin{aligned} \mathring{\varvec{\upsigma }}^q = f_s \left[ \textrm{tr}\varvec{\upsigma }^q \dot{\varvec{\upvarepsilon } } + f_v \textrm{tr}\dot{\varvec{\upvarepsilon }} \varvec{\upsigma }^q + f_a^2 \frac{\textrm{tr}(\varvec{\upsigma }^q \cdot \dot{\varvec{\upvarepsilon }})}{\textrm{tr}\varvec{\upsigma }^q} \varvec{\upsigma }^q + f_e f_a (\varvec{\upsigma }^q + \textbf{s}^q) \Vert \dot{\varvec{\upvarepsilon }} \Vert \right] \end{aligned}$$

with

28 $$\begin{aligned}&f_s = \frac{ - E_i }{3p_0(1+\nu _i)} \end{aligned}$$

29 $$\begin{aligned}&f_v = \frac{3\nu _i + f_a \sqrt{1+2\nu _i^2} }{1-2\nu _i} - \frac{f_a^2}{3} \end{aligned}$$

30 $$\begin{aligned}&f_a = \frac{\sqrt{3}\left( 3-\sin \phi \right) }{2\sqrt{2} \sin \phi } \end{aligned}$$

Notably, it can be found that the yielding criterion of Eq. (27) is $\tau = \mu _1 p^q$, which is represented by Point B in Fig. 2.

For objectivity in large deformation, the relationship between Jaumann stress rate $\mathring{\varvec{\upsigma }}^q$ and hypoplastic Cauchy stress rate $\dot{\varvec{\upsigma }}^q$ is expressed as

31 $$\begin{aligned} \dot{\varvec{\upsigma }}^q = \mathring{\varvec{\upsigma }}^q -\varvec{\upsigma }^q\cdot \dot{\varvec{\upomega }} + \dot{\varvec{\upomega }}\cdot \varvec{\upsigma }^q \end{aligned}$$

Based on Eq. (31), the quasi-static stress in Eq. (1) can be obtained by

32 $$\begin{aligned} \varvec{\upsigma }^q = \int \dot{\varvec{\upsigma }}^q \textrm{d}t \end{aligned}$$
